# Supplementary material for: Competing endogenous RNA crosstalk at system level
Source: PLoS Comput Biol. 2019 Nov 1;15(11):e1007474. doi: 10.1371/journal.pcbi.1007474 (PMC6853376; doi:10.1371/journal.pcbi.1007474)
Supplement: S1 Text — Supplementary text. (PDF) [file pcbi.1007474.s001.pdf]

Competing endogenous RNA crosstalk at system level

SUPPORTING TEXT

Mattia Miotto [1], Enzo Marinari [1] and Andrea De Martino [2,3*]
[1] *Dipartimento di Fisica, Sapienza Università di Roma, Rome (Italy)*
[2] *Soft & Living Matter Lab, CNR NANOTEC, Rome (Italy) and*
[3] *Italian Institute for Genomic Medicine, Turin (Italy)*

Contents

1. Derivation of RNA susceptibilities in generic miRNA-RNA networks 2

2. Susceptibility between distant RNA pairs 3

3. Crosstalk asymmetry 4

4. Results for degree-preserving randomized networks 5

* Corresponding author. Email: andrea.demartino@roma1.infn.it

1. DERIVATION OF RNA SUSCEPTIBILITIES IN GENERIC MIRNA-RNA NETWORKS

To derive Eq (6) of the main text, we start recasting the expressions for $[m_i]$ and $[\mu_a]$ (see Eq. (2) of main text) as

$$[m_i] = \frac{m_i^\star}{1+\displaystyle\sum_{a=1}^{M}\frac{[\mu_a]}{\mu_{ia}^0}} \equiv m_i^\star F_i \quad , \tag{1}$$

$$[\mu_a] = \frac{\mu_a^\star}{1+\displaystyle\sum_{i=1}^{N}\frac{[m_i]}{m_{ia}^0}} \equiv \mu_a^\star F_a \quad . \tag{2}$$

Using these, we immediately obtain

$$\chi_{ij} \equiv d_i\frac{\partial[m_i]}{\partial b_j} = \frac{[m_i]}{m_i^\star}\delta_{ij} + \frac{[m_i]^2}{m_i^\star}\sum_{a\in i}\frac{[\mu_a]^2}{\mu_{ia}^0\mu_a^\star}\sum_{\ell\in a}\frac{\chi_{\ell j}}{m_{\ell a}^0} \quad , \tag{3}$$

where we used the identities

$$\frac{\partial Fi}{\partial[\mu_a]} = -\frac{F_i^2}{\mu_{ia}^0} \quad , \tag{4}$$

$$\frac{\partial F_a}{\partial[m_i]} = -\frac{F_a^2}{m_{ia}^0} \quad . \tag{5}$$

Eq (8) can be re-cast in the compact form

$$\sum_{l=1}^{N}\left(\delta_{il}-W_{il}\right)\chi_{lj} = \frac{m_i}{m_i^\star}\delta_{ij} \quad , \tag{6a}$$

$$\left(\widehat{\mathbf{1}}-\widehat{\mathbf{W}}\right)\widehat{\boldsymbol{\chi}} = \mathrm{diag}\left(\frac{\mathbf{m}}{\mathbf{m}^\star}\right) \quad . \tag{6b}$$

where $\widehat{\boldsymbol{\chi}}$ is the susceptibility matrix (with elements χ_{ij}), diag $\left(\frac{\mathbf{m}}{\mathbf{m}^\star}\right)$ denotes the diagonal matrix with elements $\{m_i/m_i^\star\}$ while $\widehat{\mathbf{W}}$ is an $N\times N$ matrix with elements

$$W_{ij} \equiv \left(\widehat{\mathbf{W}}\right)_{ij} = \frac{[m_i]^2}{m_i^\star}\sum_{a\in(i\cap j)}\frac{1}{m_{ja}^0\mu_{ia}^0}\frac{[\mu_a]^2}{\mu_a^\star} \quad . \tag{7}$$

It follows that

$$\widehat{\boldsymbol{\chi}} = \left(\widehat{\mathbf{1}}-\widehat{\mathbf{W}}\right)^{-1}\mathrm{diag}\left(\frac{\mathbf{m}}{\mathbf{m}^\star}\right) \quad . \tag{8}$$

Recalling that, if all eigenvalues of $\widehat{\mathbf{W}}$ are strictly smaller than 1 in absolute values (as is easily verified numerically to the case in this study), one has $\left(\widehat{\mathbf{1}}-\widehat{\mathbf{Z}}\right)^{-1} = \sum_{n\geq 0}\widehat{\mathbf{Z}}^n$, one finds that

$$\chi_{ij} = \sum_{n\geq 0}\left(\widehat{\mathbf{W}}^n\right)_{ij}\frac{[m_j]}{m_j^\star} \equiv \sum_{n\geq 0}\chi_{ij}^{(n)} \quad . \tag{9}$$

Expressions (8) and (9) clarify an important point. While W_{ij} is different from zero only if RNAs i and j are co-regulated by at least one miRNA species, the elements of $\widehat{\mathbf{W}}^n$ are different from zero if there is at least one chain of n miRNAs joining RNAs i and j. In practice, this is what allows for crosstalk to occur even between RNAs that are not directly co-regulated, as shown explicitly within a toy model in the following section.

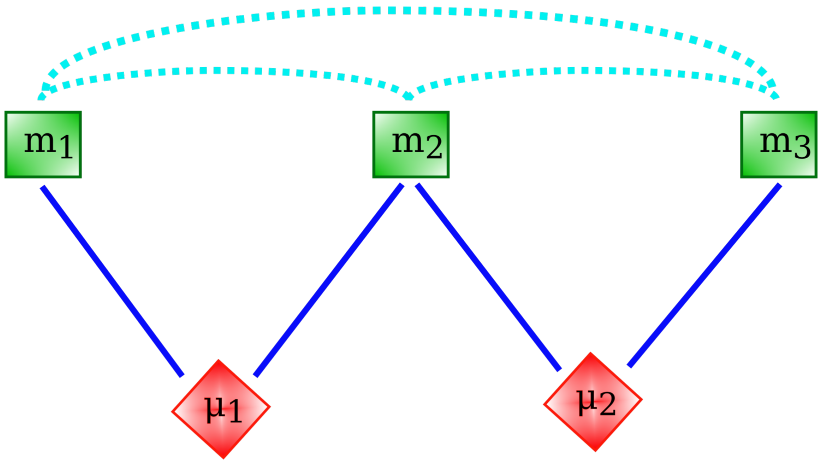

FIG. 1: **Toy miRNA-RNA network.** Two miRNA species and three RNA species interact by direct couplings represented by the continuous blue lines. Dotted lines denote instead the effective crosstalk interactions that are established between RNAs as a consequence of competition to bind miRNAs. These correspond in turn to the non-zero elements of the susceptibility matrix $\widehat{\boldsymbol{\chi}}$.

2. SUSCEPTIBILITY BETWEEN DISTANT RNA PAIRS

To show explicitly how non-zero susceptibilities can arise between pairs of RNAs connected by chains of miRNA-mediated couplings from Eq 8, we compute here the susceptibility matrix for a toy network formed by three RNA and two miRNA species, Fig 1. The $\widehat{\mathbf{W}}$ matrix for this network reads

$$\widehat{\boldsymbol{W}} = \begin{pmatrix} w_{11} & w_{12} & 0 \\ w_{21} & w_{22} & w_{23} \\ 0 & w_{32} & w_{33} \end{pmatrix} \quad . \tag{10}$$

Two elements (w_{13} and w_{31}) are nil since RNAs 1 and 3 are not co-targeted by any miRNA. Nevertheless, using (8), the susceptibility matrix turns out to be given by

$$\widehat{\boldsymbol{\chi}} = \frac{1}{\det\left(\widehat{\mathbf{1}} - \widehat{\mathbf{W}}\right)} \begin{pmatrix} (\widetilde{w}_{22}\widetilde{w}_{33} - w_{23}w_{32})\frac{m_1}{m_1^\star} & (\widetilde{w}_{33}w_{21})\frac{m_2}{m_2^\star} & (w_{21}w_{32})\frac{m_3}{m_3^\star} \\ (\widetilde{w}_{33}w_{12})\frac{m_1}{m_1^\star} & (\widetilde{w}_{11}\widetilde{w}_{33})\frac{m_2}{m_2^\star} & (\widetilde{w}_{11}w_{32})\frac{m_3}{m_3^\star} \\ (w_{12}w_{23})\frac{m_1}{m_1^\star} & (\widetilde{w}_{11}w_{23})\frac{m_1}{m_2^\star} & (\widetilde{w}_{11}\widetilde{w}_{22} - w_{12}w_{21})\frac{m_3}{m_3^\star} \end{pmatrix} \quad , \tag{11}$$

where $\widetilde{w}_{ij} = w_{ij} - 1$. Hence a non-zero susceptibility binds RNA species 1 and 3, which are connected by the chain of interactions passing through RNA 2. This connection is also evidenced by the form of the corresponding elements of $\widehat{\boldsymbol{\chi}}$. The above equation also shows explicitly that, in general, χ_{ij} and χ_{ji} are different.

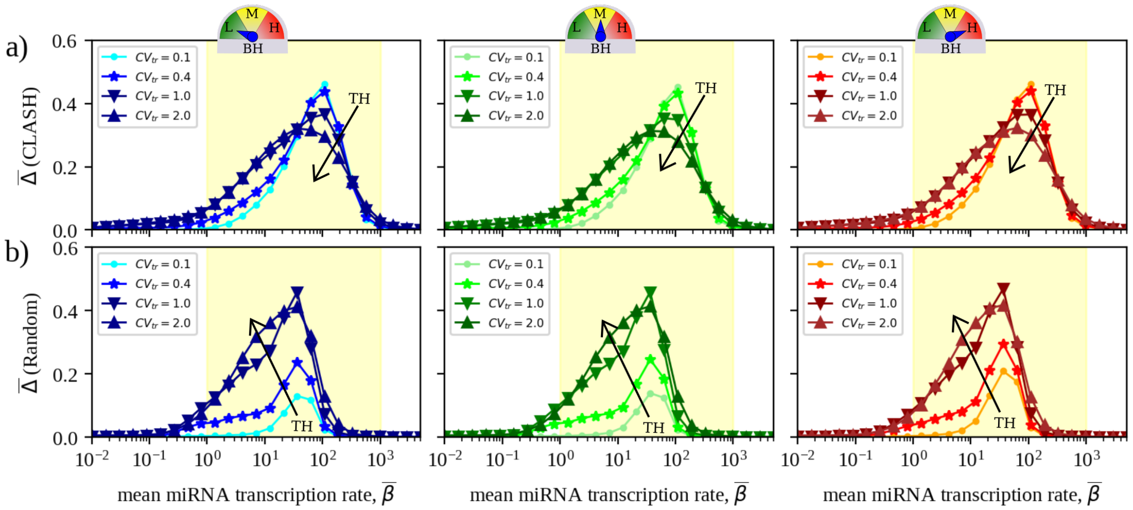

FIG. 2: **Crosstalk asymmetry in the CLASH network and its randomized counterparts.** Profile of Δ obtained **(a)** in the CLASH network, and **(b)** in its randomized version in the 3 scenarios considered for binding heterogeneity and for various degrees of transcriptional heterogeneity. Curves are averaged over 100 independent realizations of transcription rate profiles. Results for the random case are additionally averaged over 100 independent realizations of the randomization process. In each case, the standard error of the mean is equal to or smaller than the size of the markers.

3. CROSSTALK ASYMMETRY

To measure crosstalk directionality, we define the quantity

$$\Delta_{ij} = \left(\frac{\chi_{ij} - \chi_{ji}}{\chi_{ij} + \chi_{ji}} \right)^2 \quad , \tag{12}$$

such that $0 \leq \Delta_{ij} \leq 1$. In short, the closer Δ_{ij} is to zero (resp. one) the closer crosstalk between RNAs i and j is to being symmetric (resp. fully asymmetric). A global measure of asymmetry is conveniently obtained by computing the average asymmetry over all pairs of different RNAs in the network, i.e.

$$\Delta = \frac{2}{N(N-1)} \sum_{i,j>i}^{N} \Delta_{ij} \quad . \tag{13}$$

Results for this quantity are reported in Fig 2 for both the CLASH network and its randomized variant. Crosstalk asymmetry is generically larger in the susceptible regime, more pronouncedly so in the CLASH network than in its randomized version. Notably, the asymmetry profile is roughly independent of the degree of binding heterogeneity while it is only weakly modulated by transcriptional variability in the CLASH network. As seen for the mean crosstalk intensity, this state of things suggests that the way in which crosstalk asymmetry is tuned by the mean miRNA transcription rate $\overline{\beta}$ is an inherent property of miRNA-RNA networks, that is mainly encoded in their topology. The striking difference that can be seen between the behaviour of Δ in real (CLASH) and random networks (see Fig 2b) supports this intuition.

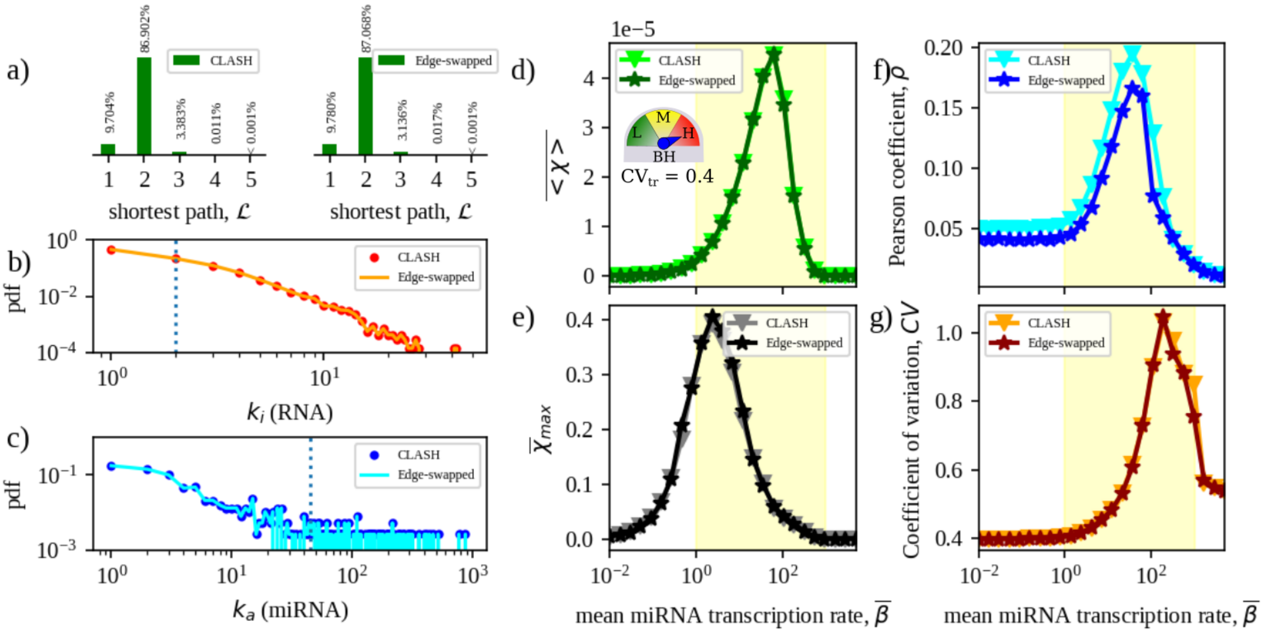

FIG. 3: **Comparison between crosstalk patterns in the CLASH network and its edge-swapped randomized versions.** **(a)** Frequency of the shortest paths for CLASH (left) and edge-swapped (right) networks. **(b, c)** Degree distributions of RNA (top) and miRNA (bottom) nodes in the CLASH and edge-swapped networks. **(d–g)** Global crosstalk descriptors for the CLASH and edge-swapped networks obtained for a degree of transcriptional heterogeneity $CV_{\text{tr}} = 0.4$ and strong binding heterogeneity as a function of the mean miRNA transcription rate $\overline{\beta}$: (d) mean susceptibility; (e) mean maximum susceptibility; (f) Pearson correlation coefficient $\overline{\rho}$ between susceptibilities and local kinetic parameters; (g) Coefficient of variation of RNA levels. Averages over 100 realizations of TH in all cases except for panel (g), where 1000 realizations were taken. In each case, the standard error of the mean is equal to or smaller than the size of the markers.

4. RESULTS FOR DEGREE-PRESERVING RANDOMIZED NETWORKS

To randomize the CLASH network while preserving the degree sequence we employ a standard edge-swapping algorithm:

1. randomly select two links ℓ_{ia} and ℓ_{jb} from the miRNA-RNA network with uniform probability;
2. swap the links, obtaining new connections ℓ_{ja} and ℓ_{ib} while keeping the inverse binding affinities (μ^0_{ia} and μ^0_{jb}) associated to RNAs i and j respectively;
3. discard the swap if it generates duplicate links or if the resulting network is not connected;
4. iterate steps 1-3 a number n of times much larger than the total number of links in the network (in our case, $n = 10^5$).

The resulting edge-swapped network has the same number of links and the same one-point statistics (i.e. the node connectivities) of the original network, while higher-order (e.g. two-node) topological correlations are lost. Numerical results were obtained by averaging over 100 independent realizations of the randomization protocol. As shown in Fig 3, the structure of randomized networks differs only slightly from that of the original CLASH network in the distribution of shortest paths between RNA species, whereas degree distributions are expectedly unchanged. In such conditions, global crosstalk descriptors are nearly identical to those obtained in the original CLASH network (panels d through g). This confirms that node degrees are the key topological determinant of the crosstalk scenario derived from the CLASH data.
