# Supplementary figures and images for: Competing endogenous RNA crosstalk at system level
Source: PLoS Comput Biol. 2019 Nov 1;15(11):e1007474. doi: 10.1371/journal.pcbi.1007474 (PMC6853376; doi:10.1371/journal.pcbi.1007474)

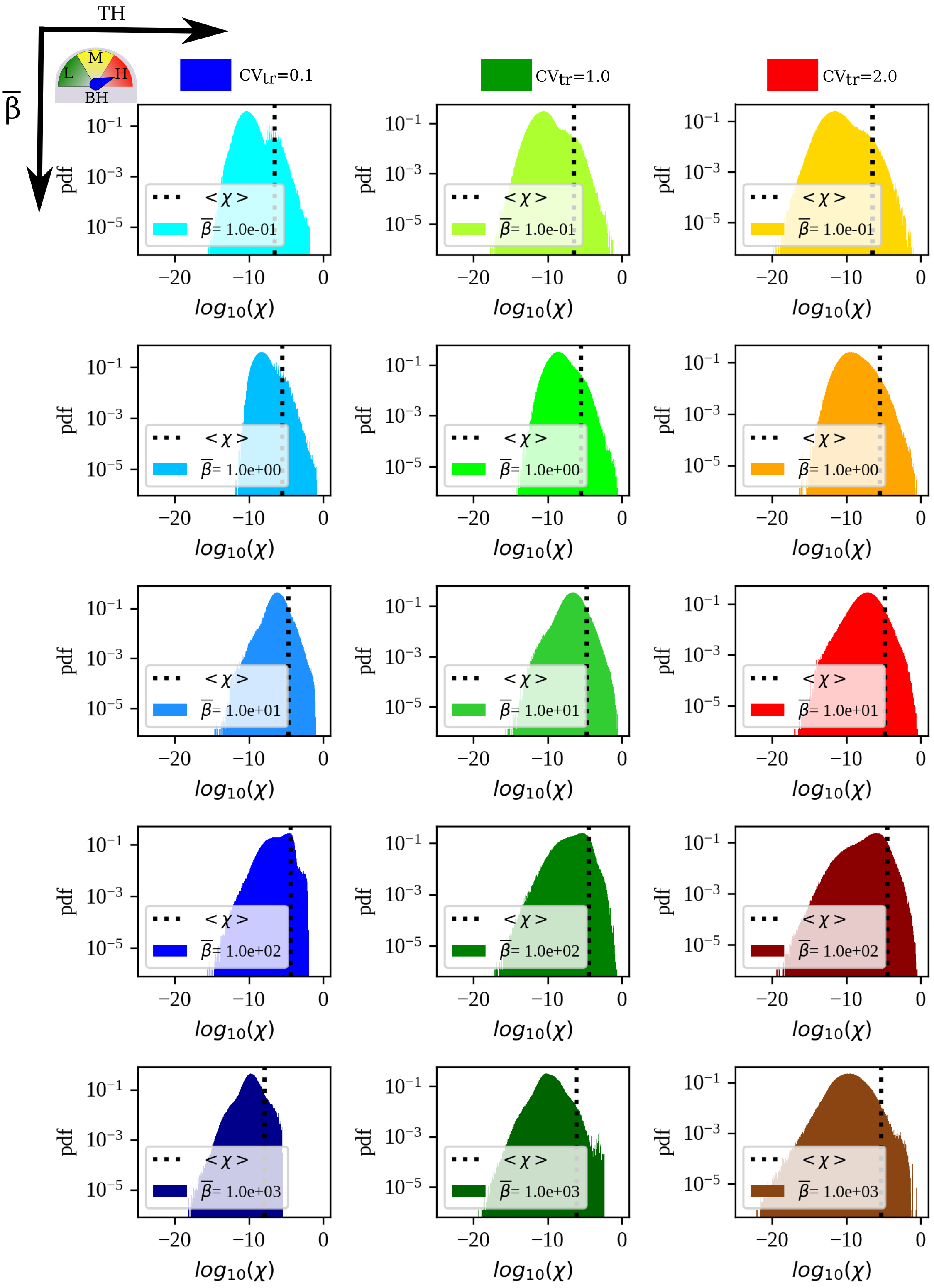

Supplement: S1 Fig — Distributions of susceptibilities between different RNA pairs for single realizations of the CLASH interactome with different values of the mean miRNA transcription rate β¯ and degrees transcriptional heterogeneity, and at fixed (high) binding heterogeneity. Note that the maximum achievable self-susceptibilities are equal to 1 (or to log10 χ = 0). (TIFF) [file pcbi.1007474.s002.tiff]

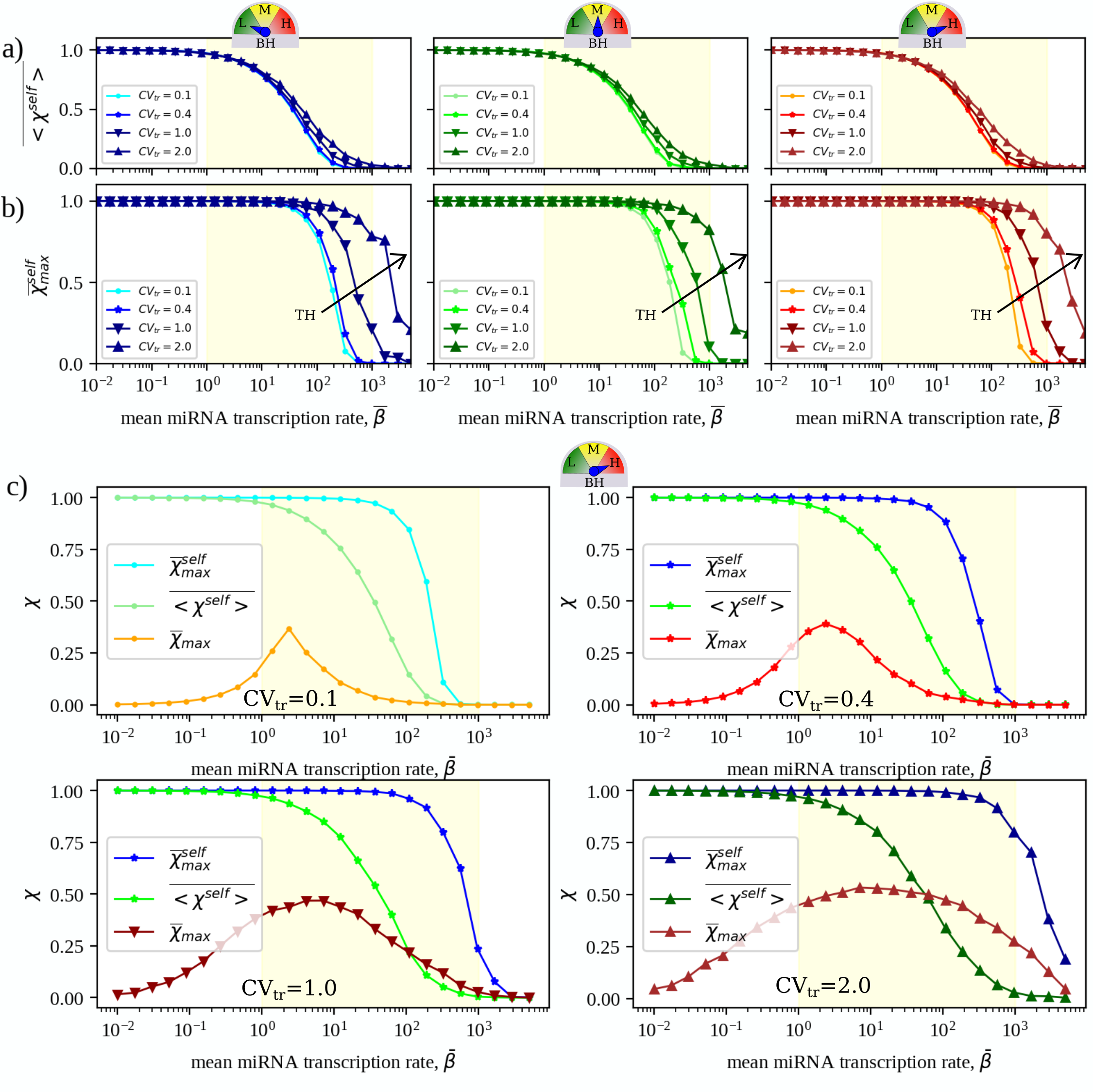

Supplement: S2 Fig — (a) Mean self-susceptibility (averaged over RNA species and over 100 independent realizations of transcriptional heterogeneity) as a function of the mean miRNA transcription rate β¯. (b) Mean maximal self-susceptibility (computed over all RNA species and averaged over 100 independent realizations of TH) as a function of the mean miRNA transcription rate β¯. Results are shown for the 3 BH scenarios considered. Parameter values are reported in Table 1 (Main Text). The yellow shaded area qualitatively marks the region where the mean susceptibility is significantly different from zero, which coincides with the susceptible regime. In each case, the standard error of the mean is equal to or smaller than the size of the markers. The self-susceptibility is maximal when miRNA levels are low, in which case the availability of free RNA molecules increases roughly linearly with the transcription rate. As β¯ increases, miRNA repression gets stronger and self-susceptibilities decrease until, at large enough β¯, RNAs are fully repressed and therefore insensitive to small changes in their transcription rates. (c) Comparison between maximum self-susceptibility (averaged over TH realizations), mean self-susceptibility (averaged over TH realizations) and χmax¯ for different degrees of TH in the high BH scenario. The intensity of crosstalk between different RNAs, measured by the latter quantity, is indeed of the same order of magnitude as self-susceptibilities. (TIFF) [file pcbi.1007474.s003.tiff]

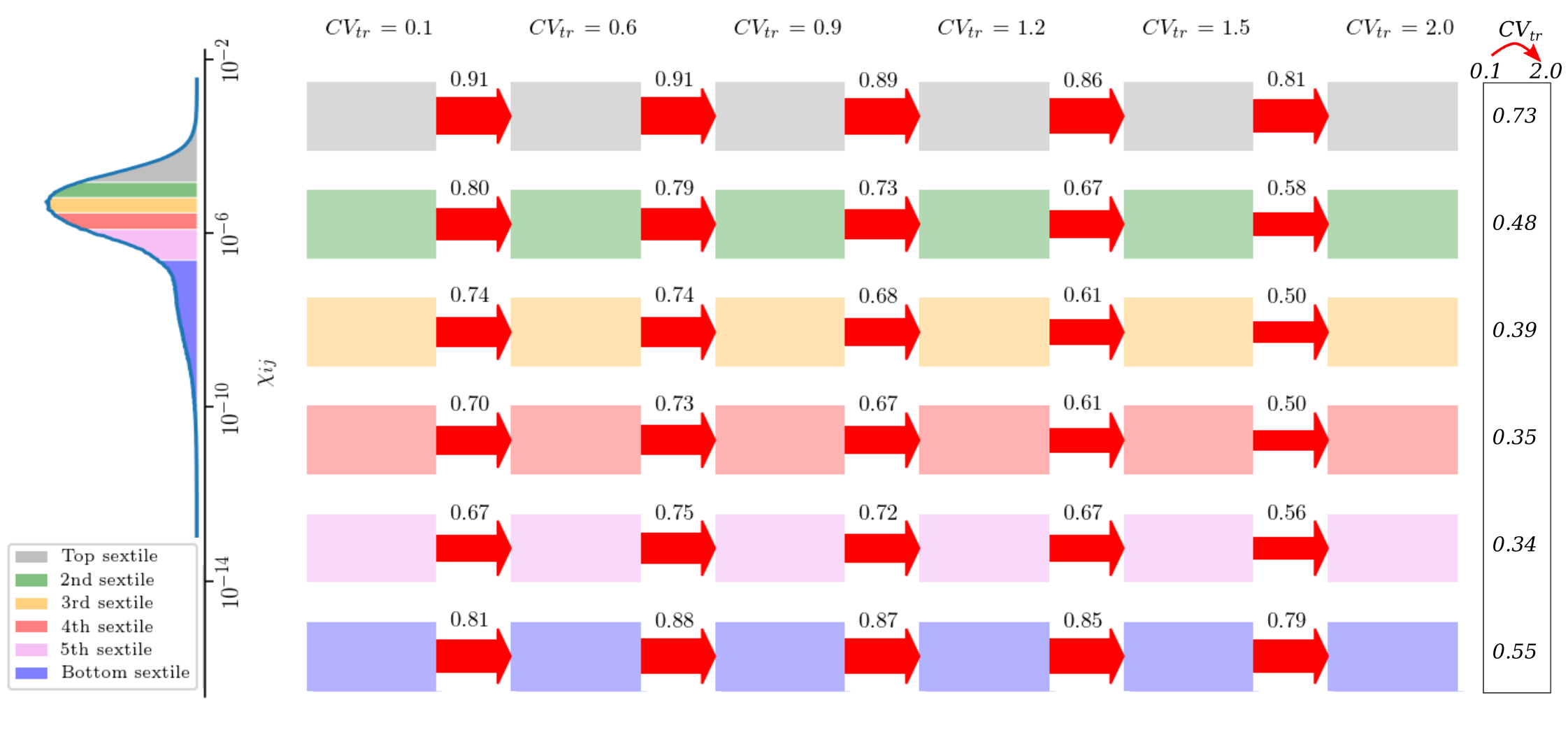

Supplement: S3 Fig — For each susceptibility sextile, we report the fraction of crosstalk interactions between distant RNAs (i.e. RNAs that do not share any miRNA regulator) that are conserved upon increasing the degree of TH. The last column reports the fraction of interactions that are conserved passing from the lowest to the highest degree of TH. Results obtained by averaging over 100 independent realizations of transcriptional heterogeneity in each case, assuming high binding heterogeneity and mean miRNA transcription rate β¯=30. Different intermediate values of β¯ return qualitatively identical scenarios. (TIFF) [file pcbi.1007474.s004.tiff]

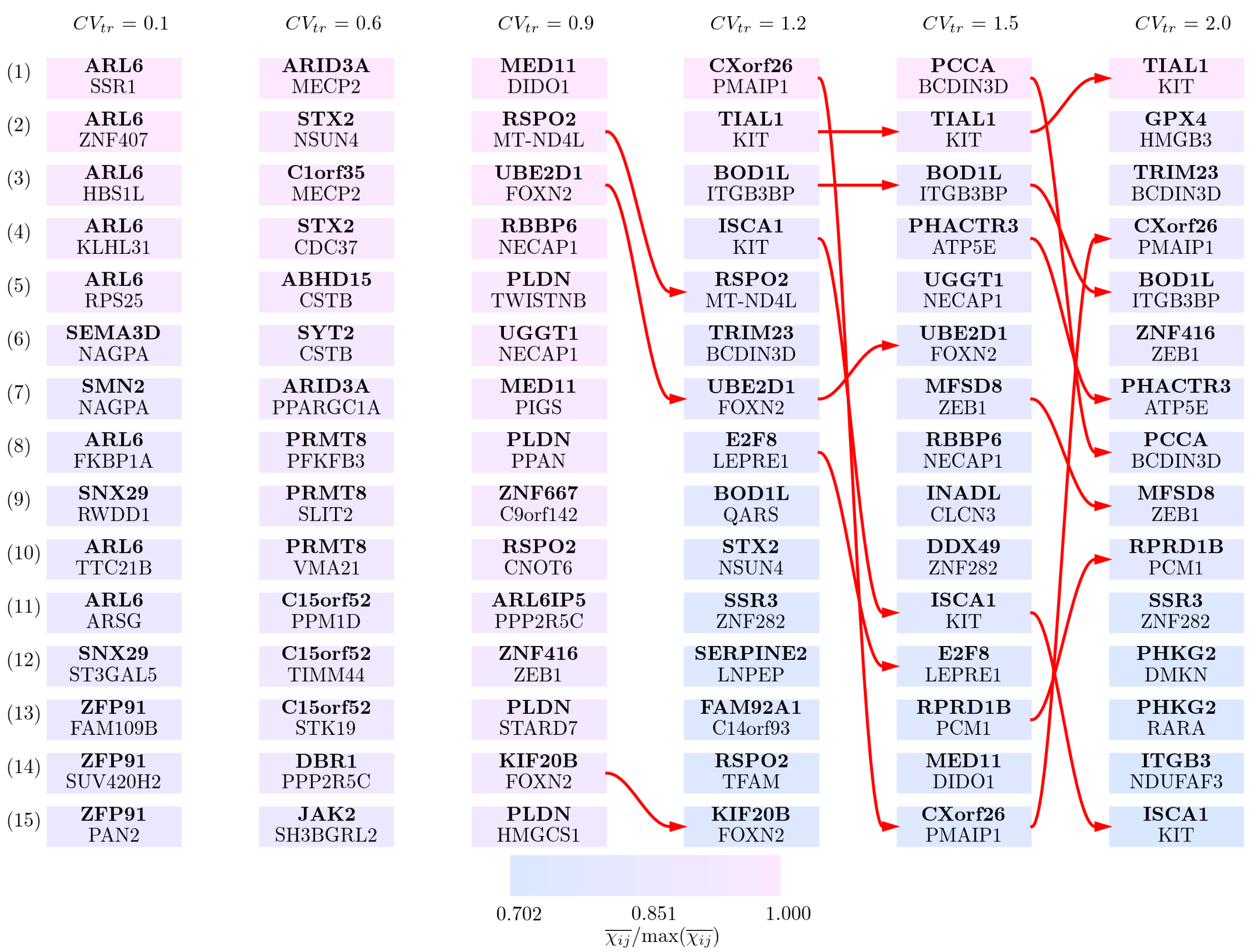

Supplement: S4 Fig — Note that involved susceptibilities (given by color code at the bottom) are of the order of the self-susceptibility. Results were obtained by averaging over 100 independent realizations of TH for each value of CVtr, assuming high BH and mean miRNA transcription rate β¯=30. (TIFF) [file pcbi.1007474.s005.tiff]

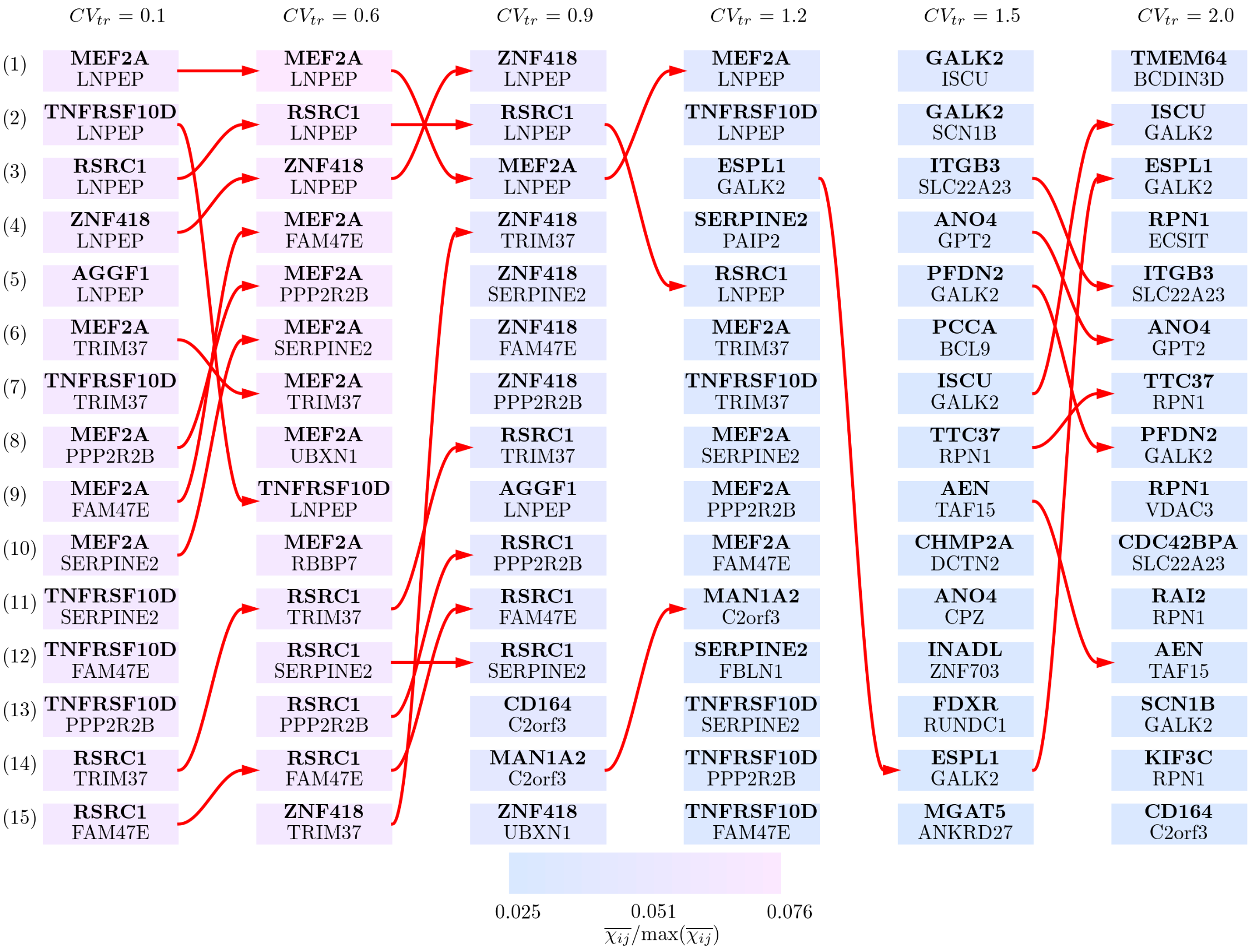

Supplement: S5 Fig — A significant degree of conservation is seen at low and high CVtr. Note that involved susceptibilities (given by color code at the bottom) are roughly 2 orders of magnitude larger than the mean susceptibility (see Main Text, Fig 2). Results were obtained by averaging over 100 independent realizations of TH for each value of CVtr, assuming high BH and mean miRNA transcription rate β¯=30. (TIFF) [file pcbi.1007474.s006.tiff]

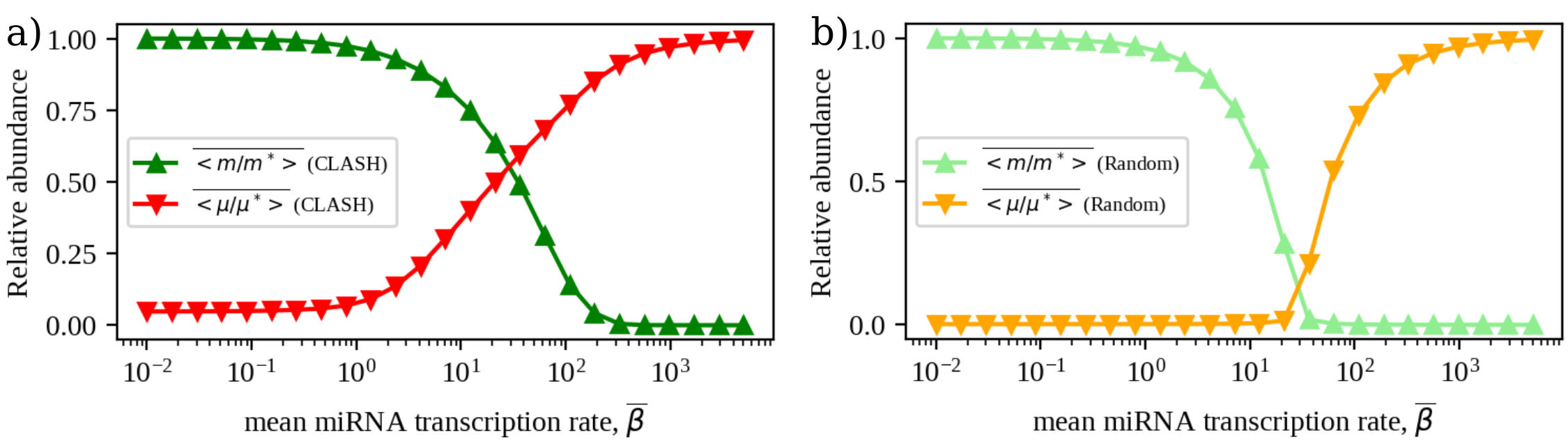

Supplement: S6 Fig — Note that the susceptible regime in the latter is narrower compared to the original CLASH network. (TIFF) [file pcbi.1007474.s007.tiff]

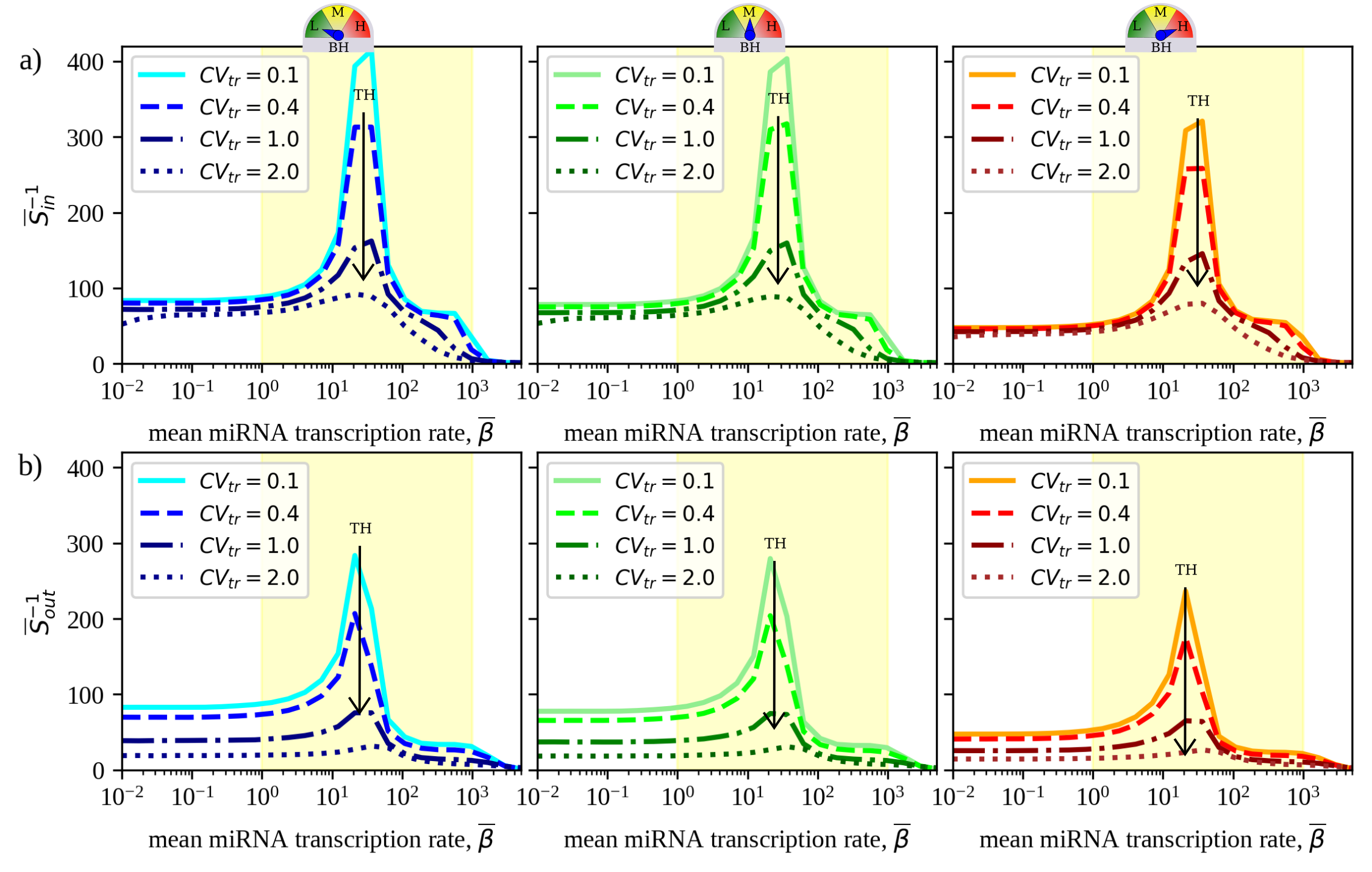

Supplement: S7 Fig — (a) Inverse of incoming and (b) outgoing selectivities as functions of β¯ for varying degrees of TH (different curves in the same panel) and BH (reported by the 3-state gauge in different panels). Curves are averaged over 100 independent realizations of transcription rate profiles and over 100 independent realizations of the randomization process. (TIFF) [file pcbi.1007474.s008.tiff]

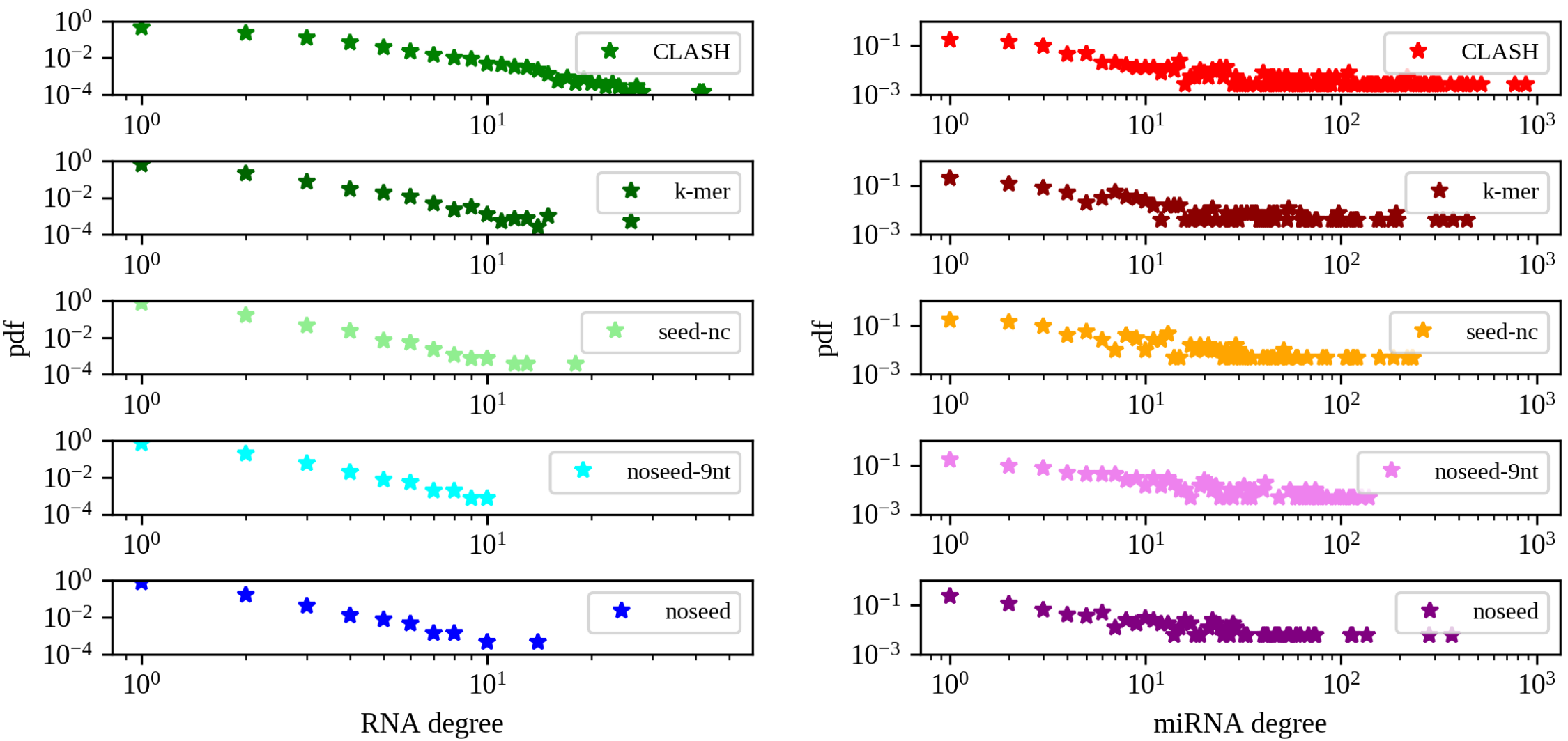

Supplement: S8 Fig — Degree distributions for RNA nodes (left) and miRNA nodes (right) are displayed for the entire network (top panels) and for the four subnetworks defined by the interaction classes considered in this work (see Main Text, Fig 1c). (TIFF) [file pcbi.1007474.s009.tiff]

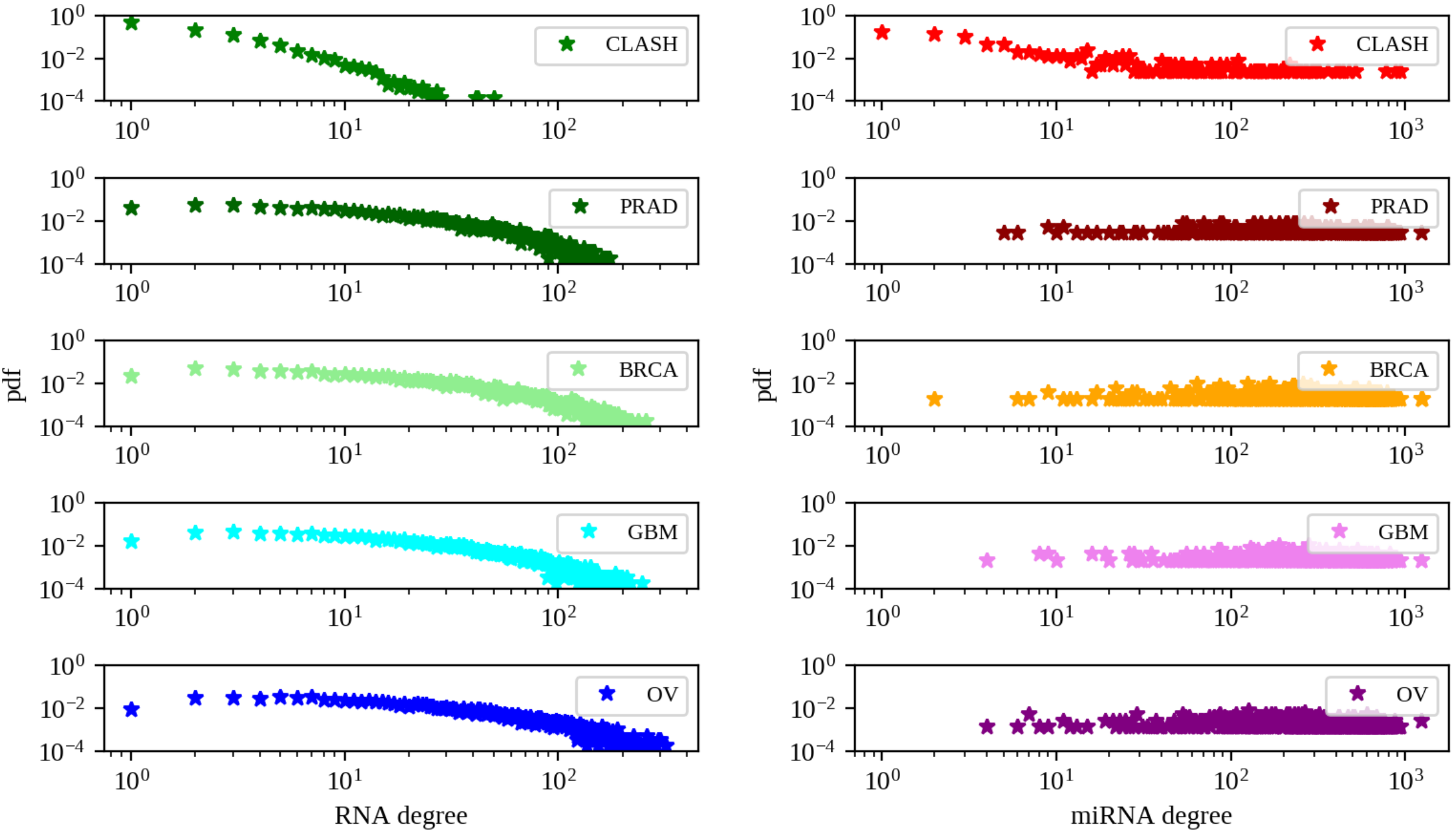

Supplement: S9 Fig — Degree distributions for RNA nodes (left) and miRNA nodes (right) representing the miRNA-RNA networks for prostate adenocarcinoma (PRAD), ovarian adenocarcinoma (OV), breast adenocarcinoma (BRCA) and glioblastoma (GBM) cells. Data from [37]. Notice that the basic characteristics of degree distributions appear to be conserved across different networks. This is possibly in line with the fact that such networks present a significant context-independent component. See [37] for a more detailed analysis. (TIFF) [file pcbi.1007474.s010.tiff]

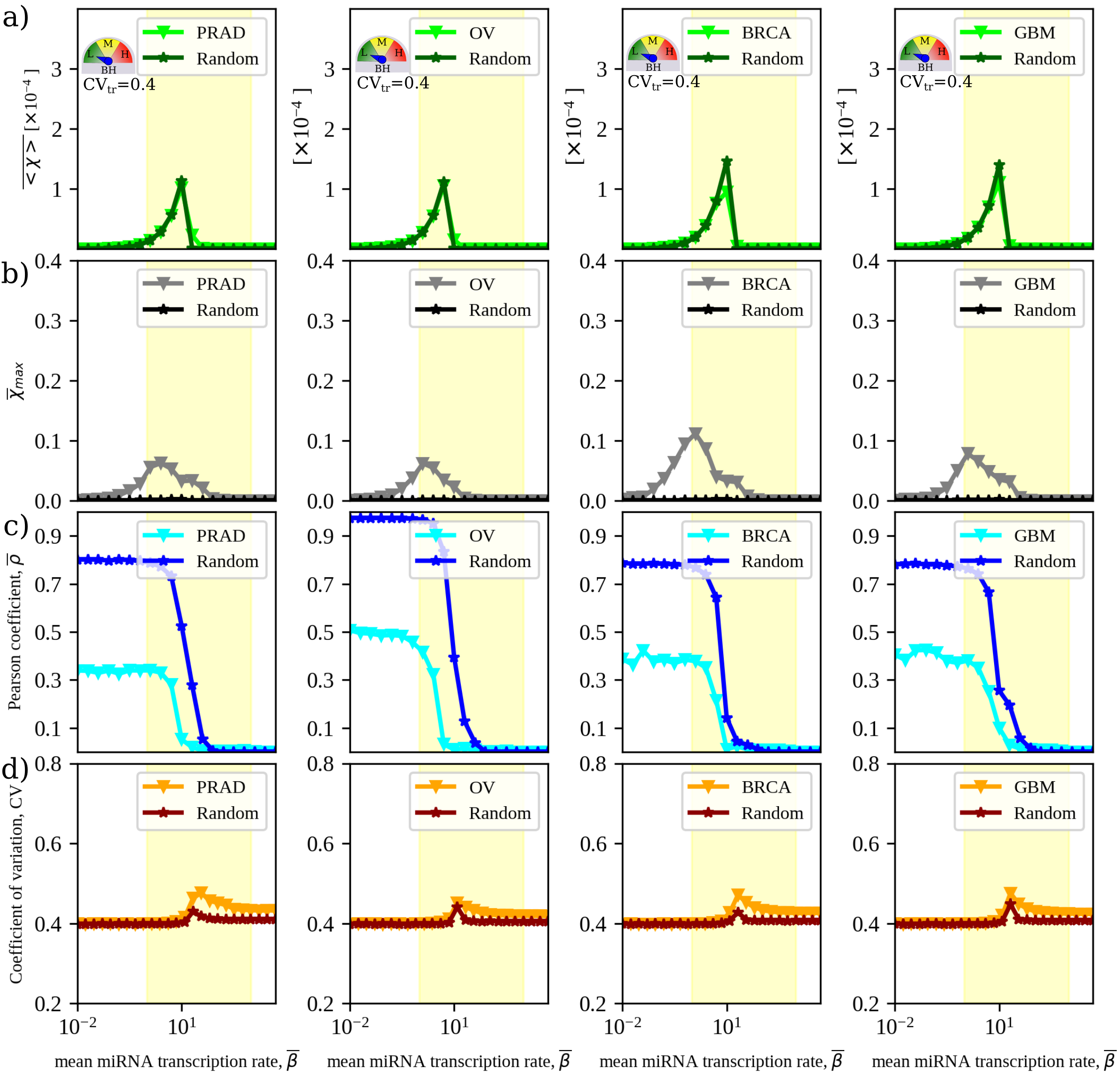

Supplement: S10 Fig — Different columns represent results obtained for prostate adenocarcinoma (PRAD), ovarian adenocarcinoma (OV), breast adenocarcinoma (BRCA) and glioblastoma (GMB) cells. (a) Mean susceptibility. (b) Maximal susceptibility. (c) Pearson correlation coefficient between susceptibilities and local kinetic parameters. (d) Coefficient of variation of RNA levels. TH was set at CVtr = 0.4 and the lowest degree of binding heterogeneity was assumed. Averages were performed over 100 realizations of TH in all cases except for panels (d), where 1000 realizations were taken. Results for other cases are qualitatively similar. (TIFF) [file pcbi.1007474.s011.tiff]
