# Supplementary material for: Competing endogenous RNA crosstalk at system level
Source: PLoS Comput Biol. 2019 Nov 1;15(11):e1007474. doi: 10.1371/journal.pcbi.1007474 (PMC6853376; doi:10.1371/journal.pcbi.1007474)
Supplement: S1 Table — Each subnetwork is obtained by selecting all links associated to the same kind of interaction occurring between the miRNA-RNA couples. If the subnetwork thus obtained is disjoint, the largest connected component was selected. The term ‘CLASH-noseed’ indicates the full CLASH network except for noseed type of interactions. (PDF) [file pcbi.1007474.s012.pdf]

Competing endogenous RNA crosstalk at system level

SUPPLEMENTARY TABLE

Mattia Miotto[1], Enzo Marinari[1] and Andrea De Martino[2,3*]
[1] *Dipartimento di Fisica, Sapienza Università di Roma, Rome (Italy)*
[2] *Soft & Living Matter Lab, CNR NANOTEC, Rome (Italy) and*
[3] *Italian Institute for Genomic Medicine, Turin (Italy)*

interaction type	nr of links	nr of RNA species	nr of miRNA species
k-mer	3624	2511	195
seed-nc	6697	3952	259
noseed-9nt	2828	2121	163
noseed	3633	2647	199
k-mer + seed-nc	10749	5262	312
CLASH - noseed	13674	6043	351
CLASH (whole)	17411	6943	383

TABLE I: Summary of the CLASH subnetwork compositions. Each subnetwork is obtained by selecting all links associated to the same kind of interaction occurring between the miRNA-RNA couples. If the subnetwork thus obtained is disjoint, the largest connected component was selected. The term 'CLASH-noseed' indicates the full CLASH network except for noseed type of interactions.

* Corresponding author. Email: andrea.demartino@roma1.infn.it
